# Patient awareness, knowledge, and acceptability of antenatal perineal massage: A single-center cross-sectional study from Saudi Arabia

**DOI:** 10.18332/ejm/194962

**Published:** 2024-11-12

**Authors:** Saeed Baradwan, Abdulrhman M. Banasser, Afaf Tawfiq, Ghaidaa Farouk Hakeem, Alya Alkaff, Bandr Hafedh, Fahad Algreisi, Taliah A. Khoja, Abdullatif Sheikh Ibrahim, Alaa Edrees

**Affiliations:** 1 Department of Obstetrics and Gynecology, King Faisal Specialist Hospital and Research Center, Jeddah, Saudi Arabia; 2 Department of Obstetrics and Gynecology, Jeddah University, Jeddah, Saudi Arabia; 3 College of Medicine, Jeddah University, Jeddah, Saudi Arabia; 4 College of Medicine, Batterjee Medical College, Jeddah, Saudi Arabia; 5 Research Department, King Faisal Specialist Hospital and Research Center, Jeddah, Saudi Arabia

**Keywords:** antenatal perineal massage, perineal tear, pregnancy, knowledge, awareness, acceptability

## Abstract

**INTRODUCTION:**

This study assessed the knowledge, awareness, and acceptability of antenatal perineal massage (APM) among pregnant women in Saudi Arabia.

**METHODS:**

This cross-sectional study included 240 pregnant women who met the predefined inclusion criteria and attended the Department of Obstetrics and Gynecology, King Faisal Specialist Hospital and Research Center, Jeddah, Saudi Arabia, from 1 October to 31 December 2023. Participants answered seven knowledge questions, classified as having good knowledge if they answered ≥4 correctly and poor knowledge if <4 were correct.

**RESULTS:**

Most women (46.25%) reported it was their first encounter with APM. Common sources of information included the internet (39.58%), physicians/midwives (7.92%), and friends/family (3.75%). Nearly half (47.5%) had heard of APM, but only 8.75% had practiced it, and 3.75% attended related classes. Only 17.5% knew that APM could be performed by the woman or her partner, while 25.83% knew it should start at 34 weeks, and 17.92% recognized the recommended duration of 5 to 10 minutes daily. Additionally, 22.5%, 15.83%, and 35.42% acknowledged APM’s benefits for labor duration, anal sphincter dysfunction, and perineal injuries, respectively. The average knowledge score was 1.39±1.84, with 84.17% classified as having poor knowledge. No significant differences were found between knowledge levels (p>0.05). Low acceptability was noted, with only 58% of patients and 37% of their partners willing to engage in APM. No significant differences in acceptability were found between poor and good knowledge groups (p>0.05).

**CONCLUSIONS:**

The study revealed low awareness, poor knowledge, and weak acceptability of APM among pregnant patients. Targeted education for patients and healthcare providers could enhance knowledge and improve maternal–fetal health outcomes.

## INTRODUCTION

The perineum is the region situated between the vaginal orifice and anus. In the context of intrapartum perineal tears, the severity of the injury is classified into four degrees, with the 3rd and 4th degrees involving damage to the anal sphincters complex and anorectal mucosa, respectively^1^. Research indicates that over 85% of women experience some form of perineal damage following vaginal childbirth, with 3rd and 4th degree tears accounting for 0.6% to 11% of these cases^1,2^.

Perineal injuries can lead to both immediate and lasting complications, which encompass issues like bleeding, pain, and infections. Furthermore, they can give rise to challenges involving incontinence, pelvic organ prolapses, disruptions in self-esteem, and heightened anxieties regarding pregnancy and childbirth^3-7^.

Hence, the prevention of such trauma stands to offer considerable advantages to many women, potentially leading to a decrease in both hospital expenses and therapeutic expenditures^8^. Considering the substantial morbidity associated with perineal trauma, several interventions have been suggested to mitigate the occurrence of this condition, such as hands-off technique, hands-on technique, warm compresses, Ritgen’s maneuver, certain breathing exercises, application of healing oils, and antenatal perineal massage^9^.

Antenatal perineal massage is a technique commonly used during pregnancy to help prepare the perineum for childbirth. An accumulating body of evidence highlights the benefits of antenatal perineal massage in mitigating the risk of severe perineal trauma, reducing the need for episiotomy, promoting wound healing, and improving APGAR scores^10,11^.

In view of the clinical benefits of antenatal massage among patients undergoing vaginal childbirth, it is important to ascertain the perceived awareness of patients toward antenatal perineal massage. In this context, only a few international studies, from United Kingdom^10^, Thailand^12^, and Brazil^13^, have examined the knowledge, attitude, and acceptability of pregnant women regarding antenatal perineal massage.

In Saudi Arabia, there has been no PubMed-indexed report addressing the awareness, knowledge, and acceptability of antenatal perineal massage among pregnant women, and this has motivated the current research. This study is essential to fill this gap in the literature, correct negative perceptions, reinforce positive attitudes, support constructive policies, and create valuable opportunities for future research.

Thepresent research presents experiences from a single-center regarding the awareness, knowledge, and acceptability of antenatal perineal massage among pregnant women in Saudi Arabia. We hypothesized that the surveyed participants would exhibit limited knowledge, low awareness levels, and reduced acceptability of antenatal perineal massage.

## METHODS

### Study design, setting and participants

The research was a cross-sectional, observational study, conducted at the maternal–fetal clinic at the Department of Obstetrics and Gynecology, King Faisal Specialist Hospital and Research Center, Jeddah, Saudi Arabia, from 1 October to 31 December 2023. The inclusion criteria were consenting pregnant women attending routine antenatal care for vaginal childbirth at the maternal–fetal clinic, who completed survey. The exclusion criteria included all pregnant women who did not provide consent, attended the maternal-fetal medicine clinic outside the predefined time frame, or failed to fill in the survey questionnaire completely.

A sample size calculator was used to compute the essential sample for the research. Based on a 5% margin of error, confidence level of 95%, 50% response distribution, and a population size of 600 patients (average is 50 patients per week), the calculated sample size was roughly 235 participants. The targeted subjects included all participants without any sociodemographic restrictions. Participants with missing data were excluded. Convenience non-probability sampling was employed, with participants chosen for inclusion based on their easy accessibility to the researchers, as they were given direct access to the online questionnaire.

### Ethical considerations

The research was granted approval by the related Institutional Review Board (IRB) and Research Advisory Council (RAC approval identifier: 2023-129, October 2023). Importantly, the study relied on an anonymous survey, and participation was on a volunteering basis after signing an informed consent. Patients were assured with regard to the confidentiality of data and its anonymity. The research did not present more than minimal risk of harm to the subjects. The subjects were provided with pertinent information after participation, whenever appropriate.

### Data sources, management, and variables

The questionnaire was conducted in the office of maternal–fetal medicine clinics through Google Form Document with the help of clinical residents and social workers not involved in the study. The estimated completion time was approximately 10 minutes. The anonymous data were electronically gathered and organized in Microsoft Excel afterward for analysis. The principal investigator was the guarantor of data storage and concealment.

The survey was tentatively constructed in a way similar to a published article^10^. However, some new questions thought significant by the authors, we real so considered owing to reasons pertaining to emerging literature and specific gaps identified in the original questionnaire. Afterward, the survey was examined for content and face validity^14^. Validity was assessed through pilot testing, in which the survey was administered to 10 participants to ensure that the questions were clearly understood. No modifications were made following the pilot testing, and the results from this phase were not included in the final analysis.

In this study, the approach outlined by Labrecque et al.^15^ was adopted as the standard technique. This method involves a daily massage lasting 5 to 10 minutes, starting from 34 weeks of gestation and continuing until delivery. The questionnaire was divided into four main parts: 1) baseline sociodemographics (9 questions); 2) awareness of antenatal perineal massage (3 questions); 3) knowledge about antenatal perineal massage (7 questions); and 4) acceptability of antenatal perineal massage (2 questions).

The sociodemographics included age (18–39 vs ≥40 years), number of previous deliveries (≤3 vs≥4), mode of most recent delivery (vaginal, ventose, cesarean section, or first pregnancy), number of current fetuses in current pregnancy (1 vs≥2), highest level of education (≤secondary school vs>secondary school), number of individuals in family (≤5 vs ≥6), monthly income (<15000 vs ≥15000 SAR), coexisting morbidity (no vs yes), and main source of knowledge on antenatal perineal massage. The awareness questions were configured in a yes/no format. The knowledge questions were presented in both multiple-choice format, with one correct answer, and a yes/no format, also with a single correct answer. For the knowledge questions (n=7), participants received 1 point for each correct answer and 0 points for incorrect answers. The total score was calculated by summing these points. Participants were classified as having good knowledge if they answered ≥4 questions correctly and as having poor knowledge if they answered <4 correctly. This cut-off was based on the authors’ judgment that a threshold of more than 50% correct answers is appropriate and defines ‘good’ knowledge. The acceptability questions were rated using a 5-point Likert scale, with the following values: 1=strongly disagree, 2=disagree, 3=neutral, 4=agree, and 5=strongly agree.

### Statistical analysis

Numerical data were summarized as mean ± standard deviation (range: minimum–maximum), and analyzed using the Student’s t-test. Categorical data were summarized as numbers and percentages and analyzed using the chi-squared and/or Fischer’s exact test, as appropriate. We assessed whether the level of knowledge (good vs poor) was influenced by various sociodemographics. The differences between patients with good and poor knowledge were summarized as mean differences with 95% confidence intervals (CI). Additionally, we examined if there were differences in the acceptability scores between patients with good versus poor knowledge. Data were analyzed using the SPSS software, version 29.0 for Windows. Two-tailed p<0.05 established statistical significance.

## RESULTS

Table 1 summarizes the sociodemographic and clinical characteristics of the surveyed participants. A total of 240 patients took part in the research. Most patients were aged 18–39 years (90.42%), had ≤3 previous deliveries (88.75%), were carrying single fetuses in their current pregnancies (93.75%), and had an education level of at least high school or higher (90%). Additionally, nearly three-quarters of the patients reported monthly incomes below the national average of 15000 SAR (72.5%) and did not have any comorbidities (75%). When asked about their primary source of information on antenatal perineal massage, most patients (46.25%) indicated that it was their first time hearing about this practice. The three most frequently reported sources of information included the Internet (39.58%), physicians/midwives (7.92%), and friends/family (3.75%).

**Table 1:** Sociodemographic and clinical characteristics of the surveyed participants

*Characteristics*	*n (%)*
**Age** (years)	
18–39	217 (90.42)
≥40	23 (9.58)
**Previous deliveries**	
≤3	213 (88.75)
≥4	27 (11.25)
**Mode of most recent delivery**	
Normal vaginal	91 (37.92)
Ventouse vaginal	3 (1.25)
Cesarean section	73 (30.42)
First pregnancy	73 (30.42)
**Number of individuals in the family**	
≤5	189 (78.75)
≥6	51 (21.25)
**Number of embryos in current pregnancy**	
1	225 (93.75)
≥2	15 (6.25)
**Education level**	
≤High school	24 (10.00)
>High school	230 (90.00)
**Monthly income** (SAR)	
<15000	174 (72.50)
≥15000	66 (27.50)
**Comorbidities**	
No	180 (75.00)
Yes	60 (25.00)
**Main source of information on antenatal perineal massage**	
First time hearing about it	111 (46.25)
Pregnancy classes	0 (0)
Physicians/midwives	19 (7.92)
Books	4 (1.67)
Friends/family	9 (3.75)
Journals	2 (0.82)
Shopping malls	0 (0)
Internet	95 (39.58)
Pamphlets	0 (0)

SAR: 1000 Saudi Arabian Riyal about US$270.

Table 2 summarizes patients’ awareness of antenatal perineal massage in a yes/no format. Nearly half of the participants (47.5%) reported having heard of antenatal perineal massage. However, very few patients indicated that they had actually performed the practice (8.75%) or attended any related classes or workshops (3.75%).

**Table 2:** Awareness of antenatal perineal massage among the surveyed participants

*Awareness questions*	*n (%)*
**Have you heard of antenatal perineal massage before?**	
No	126 (52.50)
Yes	114 (47.50)
**Have you performed the antenatal perineal massage before?**	
No	219 (91.25)
Yes	21 (8.75)
**Have you attended any related lessons or workshops for antenatal perineal massage before?**	
No	231 (96.25)
Yes	9 (3.75)

Table 3 summarizes patients’ knowledge of antenatal perineal massage using both single-answer multiple-choice questions and yes/no formats. Only 17.5% of patients correctly identified that antenatal perineal massage can be performed by the pregnant woman and/or her partner. Furthermore, just 4.58% recognized that oils, warm compresses, and lubricants can be used during the practice. Only 25.83% of patients accurately detailed that antenatal perineal massage should begin at 34 weeks of gestation until delivery, while 17.92% identified the recommended duration of 5 to 10 minutes daily. Additionally, only 22.5%, 15.83%, and 35.42% of patients correctly acknowledged the scientific evidence that antenatal perineal massage can lessen the length of the second stage of labor, anal sphincter dysfunction, and the occurrence of episiotomy or related perineal injuries, respectively. For all patients, the composite knowledge mean score was 1.39 ± 1.84 (range: 0–7). According to the composite knowledge score, the majority of patients were categorized as having poor knowledge (n=202; 84.17%), while only a few were classified as having good knowledge (n=38; 15.83%). No significant difference was noted in the knowledge scores between patients with poor and good knowledge levels (all p>0.05, data not shown).

**Table 3:** Knowledge of antenatal perineal massage among the surveyed participants

*Knowledge questions*	*n (%)*
**Who can perform the antenatal perineal massage during pregnancy?**	
Myself	56 (23.33)
Husband	5 (2.08)
Myself and husband	42 (17.50)
I don’t know	137 (57.08)
**What substances can be used during antenatal perineal massage?**	
Oils	43 (17.92)
Warm compressors	1 (0.42)
Lubricants	5 (2.08)
Oils and warm compressors	5 (2.08)
Warm compressors and lubricants	15 (6.25)
Oils and lubricants	0 (0)
Olis, warm compressors, and lubricants	11 (4.58)
I don’t know	160 (66.67)
**What is the recommended time of antenatal perineal massage daily?**	
<20 weeks of gestation	8 (3.30)
From 20 to 34 weeks of gestation	10 (4.17)
From 34 weeks of gestation until delivery	62 (25.83)
I don’t know	160 (66.67)
**What is the recommended duration of antenatal perineal massage daily?** (minutes)	
<5	14 (5.83)
5–10	43 (17.92)
>10	4 (1.67)
I don’t know	179 (74.58)
**Is there strong scientific evidence that antenatal perineal massage reduces the second-phase of labor?**	
No	9 (3.75)
Yes	54 (22.50)
I don’t know	177 (73.75)
**Is there strong scientific evidence that antenatal perineal massage reduces anal sphincter dysfunction?**	
No	8 (3.33)
Yes	38 (15.83)
I don’t know	194 (80.83)
**Is there strong scientific evidence that antenatal perineal massage reduces episiotomy and perineal tears?**	
No	4 (1.67)
Yes	85 (35.42)
I don’t know	151 (62.92)

Table 4 presents the acceptability of antenatal perineal massage. The findings indicate low levels of acceptability, with only 58% of patients and 37% of their partners expressing a willingness to engage in this practice during pregnancy (i.e. those who agreed or strongly agreed).The Likert mean scores for the acceptability of patients and partners to perform antenatal perineal massage during pregnancy were 3.5 ± 1.1 (range: 1–5) and 3.06 ± 1.11 (range: 1–5), respectively. No significant difference was noted between individuals with poor and good knowledge levels regarding the acceptability of patients (mean difference= -0.56; 95% CI:-0.94 – -0.18, p=0.998) and partners (mean difference= -0.27; 95% CI:-0.66–0.12, p=0.915) to perform antenatal perineal massage during pregnancy.

**Table 4:** Acceptability of antenatal perineal massage among the surveyed participants

*Acceptability items*	*n (%)*
**I have the acceptability to perform antenatal perineal massage during pregnancy**	
Strongly disagree	21 (8.75)
Disagree	15 (6.25)
Neutral	64 (26.67)
Agree	102 (42.50)
Strongly agree	38 (15.83)
**My husband has the acceptability to perform antenatal perineal massage during pregnancy**	
Strongly disagree	28 (11.67)
Disagree	37 (15.42)
Neutral	86 (35.83)
Agree	70 (29.17)
Strongly agree	19 (7.92)

## DISCUSSION

This research was conducted at a single center in Saudi Arabia to explore the awareness, knowledge, and acceptability of antenatal perineal massage. A total of 240 pregnant patients took part in the research. The findings highlight that most patients had limited awareness of antenatal perineal massage, and the majority (84%) demonstrated poor knowledge. Additionally, the findings revealed low levels of acceptability for antenatal perineal massage, with only 58% of patients and 37% of their partners expressing willingness to engage in this practice during pregnancy. To the best of our knowledge, this study is novel as it represents the second research effort conducted in Saudi Arabia to examine patients’ awareness, knowledge, and acceptance of antenatal perineal massage.

The results of this study are concerning, revealing that most patients had limited awareness of antenatal perineal massage, with a staggering 84% demonstrating poor knowledge of the practice. Such figures highlight a significant gap in education surrounding antenatal care, which is crucial for enhancing maternal health outcomes. The low levels of acceptability, with only 58% of patients and 37% of their partners expressing willingness to engage in this practice, further emphasize the need for improved educational initiatives. This lack of awareness and understanding could prevent many pregnant women from benefiting from the advantages of antenatal perineal massage. It is essential to address this issue by providing targeted educational programs that inform both patients and their partners about the benefits and techniques of perineal massage. Rectifying this matter will not only empower expectant mothers but also contribute to better health outcomes during labor and delivery^10,11^.

Feedback from healthcare professionals engaged in antenatal care, such as midwives and obstetricians, is equally vital. In our study, only 8% of patients identified physicians and midwives as sources of information about antenatal perineal massage. This figure is quite discouraging and highlights the urgent need for these healthcare providers to adopt more proactive roles in patient education. Midwives, in particular, are essential for educating patients during pregnancy and are often directly involved in their care throughout the childbirth process. Furthermore, <35% of patients were aware of the scientific evidence supporting antenatal perineal massage in curtailing the extent of the second phase of labor, minimizing anal sphincter damages, and decreasing the necessity for episiotomy. This statistic underscores the insufficient knowledge among healthcare providers and/or their failure to effectively communicate this important information to patients, preventing them from being informed and empowered to practice antenatal perineal massage.

Various factors contribute to the possibility of perineal injuries during vaginal labor. Such examples comprise being a first-time mother, advanced maternal age, undergoing operative delivery through forceps or vacuum extraction, having a larger-than-average baby, experiencing an extended second phase of labor, and assuming certain positions during labor^16-19^.

A meta-analysis of 11 clinical trials showed that women who underwent antenatal perineal massage exhibited a noteworthy reduction in the occurrence of episiotomies, perineal tears (particularly pronounced in the case of 3rd and 4th degree perineal injuries). Additionally, the antenatal perineal massage group showed improved wound healing and reduced perineal discomfort. Besides, the intervention culminated in a reduction in the duration of the second phase of labor, a reduction in anal incontinency, and noteworthy enhancements in Apgar scores at both 1 and 5 minutes. Collectively, the research concluded that engaging in antenatal perineal massage is connected to a decreased likelihood of experiencing severe perineal injury and postpartum complications^11^.

In view of the clinical benefits of antenatal massage among patients undergoing vaginal childbirth, it is critically important to scrutinize the perceived awareness of patients toward antenatal perineal massage. Within these lines, apart from our local study, only a few international studies, from United Kingdom^10^, Thailand^12^, and Brazil^13^, examined the knowledge, attitude, and acceptability of pregnant women regarding antenatal perineal massage^10^.

Ismail et al.^10^ conducted a study to evaluate the acceptability of antenatal massage among British pregnant women and their awareness of the technique. An anonymous questionnaire was given to mothers after their first delivery, yielding 113 responses over four months. Results showed that 61% found the practice acceptable, 26% felt embarrassed about it, and 57% were comfortable having their partner perform it. Awareness was low: 37% had heard of the practice, 10% knew it should start at 34 weeks, 12% recognized it should last 5–10 minutes, and 30% understood it should be done daily. This indicates a need for increased education and support to promote antenatal perineal massage among first-time mothers^10^.

Meeprom et al.^12^ conducted a cross-sectional study between July 2021 and June 2022 in Bangkok, Thailand. The study aimed to assess the knowledge, attitudes, and acceptability of antenatal perineal massage among Thai women at ≥22 weeks of gestation. Participants completed a self-administered questionnaire, and in-depth interviews were conducted with pregnant patients not desiring antenatal perineal massage. Results revealed that out of the 144 enrolled pregnant women, 83% exhibited a favorable attitude towards antenatal perineal massage. In terms of knowledge, 15% participants were aware of the practice, 32% knew it should be initiated after reaching 34 weeks of gestational age, 36% understood that the massage should last for 5–10 minutes, and 26% recognized that it ought to be completed daily. Factors linked to the adoption of antenatal perineal massage included previous curiosity in perineal massage and a belief in the beneficial impact of the practice in enabling vaginal childbirth. On the other hand, reasons for declining antenatal perineal massage comprised never having heard of it, concerns about pregnancy difficulties, anxiety of discomfort, perceiving it as an ineffective procedure, and having experienced successful previous vaginal deliveries. In conclusion, the study identified a high level of acceptability for antenatal perineal massage among the participants. As a result, the implementation of this scheme should be regularly clarified and presented to pregnant women to potentially lessen the occurrence of severe perineal injury and postpartum aftermaths^12^.

Gondim et al.^13^ carried out a cross-sectional study, involving Brazilian women, within the first 72 hours following vaginal childbirth, and who had expressed a desire for and experienced vaginal birth. The study intended to judge the knowledge, attitudes, and practices (KAP) related to orienting the pelvic floor muscles for labor. Sociodemographic, clinical, and obstetric information was gathered from healthcare records. A 15-question survey was employed to gauge participants’ KAP, categorizing knowledge as poor, average, or good. The survey involved 326 women. Out of these, 51% women demonstrated poor knowledge about PFM preparation. Merely 4% individuals reported seeking information on how to avoid perineal injury, and only 4% stated that they had engaged in pelvic floor muscle training during their gravidity (including perineal massage). Multivariate analysis indicated that individuals with low levels of education exhibited poor knowledge. In conclusion, the study revealed inadequate knowledge, attitudes, and practices concerning pelvic floor muscle preparation for labor. Addressing health schooling concerning pelvic floor muscle care during gravidity and after birth is crucial, especially among pregnant women who are younger, possess low level of education, and have limited salary^13^.

Metwally and Attas investigated the awareness and acceptance of antenatal perineal massage among Saudi mothers^20^. The study utilized a cross-sectional design, surveying mothers in Saudi Arabia who had delivered their first child. An electronic questionnaire assessed their knowledge and acceptance of perineal massage, as well as details about their previous pregnancies and deliveries. Of the 113 women who participated, the average age was 33.58 years, with approximately 69% unaware of perineal massage and 62.8% unfamiliar with its application; 58.4% reported not performing pelvic floor exercises during pregnancy. While most participants were receptive to the idea of perineal massage and accepted that their partners could assist, overall awareness was low, despite a relatively high level of acceptance of the practice^20^. Our current study builds upon the previously referenced one^20^, featuring a larger sample size and a focus on evaluating awareness levels.

Raising mindfulness of antenatal perineal massage must preferably be a portion of a broader strategy to endorse pelvic floor fortification during pregnancy and labor. Antenatal perineal massage should be practiced in conjunction with antenatal pelvic floor exercises, as both lead to the safeguarding of pelvic floor health^21^. Likewise, applying warm compressors to the perineum during labor has been demonstrated to decrease the frequency of anal sphincter damages^9^. Additionally, patient information and education must be designed to enhance the adoption of antenatal perineal massage. This practice necessitates motivation and commitment, both of which are crucial since it requires daily engagement without immediate, visible benefits^10^. In a randomized control trial, 161 primiparous women were assigned to either the smartphone website group (n=81) or the leaflet group (n=80)^22^. The primary outcome, the continuance rate of antenatal perineal massage practiced three times a week over three weeks, showed rates of 51.1% for the website group and 51.0% for the leaflet group, with no significant differences between them. Additionally, secondary outcomes related to perineal massage evaluation, childbirth self-efficacy, and satisfaction did not differ significantly between the groups, indicating that both methods were more effective than no instructions at all^22^.

### Strengths and limitations

This study has several strengths, including being the second research conducted in Saudi Arabia to examine patients’ awareness, knowledge, and acceptability of antenatal perineal massage. Additionally, the study benefits from a relatively large sample size of 240 participants, which enhances the robustness of the findings. However, it also has limitations that warrant acknowledgment. One shortcoming is that it was conducted at a single hospital, which means the findings may not be generalizable to the national population. Additionally, since the data were collected through self-reported questionnaires, there is a risk of overestimation and underestimation, which may compromise data accuracy. Furthermore, asking participants about past experiences raises concerns about recall bias, which can affect the reliability of the information gathered. The cross-sectional nature of the data collection restricts the study’s ability to establish causal relationships, allowing only for the identification of associations. The decision not to control for confounding variables may limit the validity of the findings, as unaccounted factors could influence the results.

### Implications

Forth coming investigation should concentrate on extending this study by including multiple centers across Saudi Arabia to augment the generalizability of the conclusions. Moreover, longitudinal investigations could offer deeper understandings into the long-term effects of antenatal perineal massage on maternal-neonatal outcomes. Implementing targeted educational interventions for healthcare providers, particularly midwives and obstetricians, could also improve patient awareness and knowledge. Furthermore, exploring the barriers to practicing antenatal perineal massage among pregnant women and their partners could inform strategies to increase its acceptability. Lastly, integrating qualitative methods could enrich our understanding of patients’ perspectives and experiences regarding antenatal care practices, paving the way for more effective education and support initiatives.

## CONCLUSIONS

The current research found low level of awareness, poor knowledge, and weak acceptability of pregnant patients toward antenatal perineal massage. Implementing targeted educational interventions for patients and healthcare providers, especially midwives and obstetricians, could enhance patient awareness and knowledge. Addressing these gaps is crucial for improving maternal health outcomes.

## Data Availability

The data supporting this research are available from the authors on reasonable request.
